# Effect of Ba and Pb dual doping on the thermoelectric properties of BiCuSeO ceramics

**DOI:** 10.1016/j.dib.2018.09.088

**Published:** 2018-10-03

**Authors:** Bo Feng, Guangqiang Li, Zhao Pan, Yanhui Hou, Chengcheng Zhang, Chengpeng Jiang, Jie Hu, Qiusheng Xiang, Yawei Li, Zhu He, Xi'an Fan

**Affiliations:** a The State Key Laboratory of Refractories and Metallurgy, Wuhan University of Science and Technology, Wuhan 430081, China; b Key Laboratory for Ferrous Metallurgy and Resources Utilization of Ministry of Education, Wuhan University of Science and Technology, Wuhan 430081, China

## Abstract

Besides the thermoelectric properties, mechanically robust is also very important for applications in TEGs. Up to now, no studies have been reported to investigate the mechanical properties of BiCuSeO oxyselenides. In this work, the results of hardness test of pristine and Ba/Pb doped BiCuSeO are presented here. These data may help to further evaluate the mechanical properties of BiCuSeO based ceramics.

**Specifications table**

**Table:** 

Subject area	Physics
More specific subject area	BiCuSeO based thermoelectric materials
Type of data	Text file, figure
How data was acquired	hardness-testing device (HV-10008, China)
Data format	Raw data
Experimental factors	Brief description of any pretreatment of samples
Experimental features	Brief experimental description
Data source location	Wuhan, China
Data accessibility	The data is with this article, not in public repository.

**Value of the data**

• Mechanical properties, like hardness, have a great influence on the processability of thermoelectric materials, so it is necessary to explain it.
• There has been no previous literature to study the hardness of BiCuSeO, so it is necessary to investigate.
• Upon Ba/Pb doping, the change of hardness is obvious, so it is necessary to explain.

## 1 Data

**Fig. 1:**
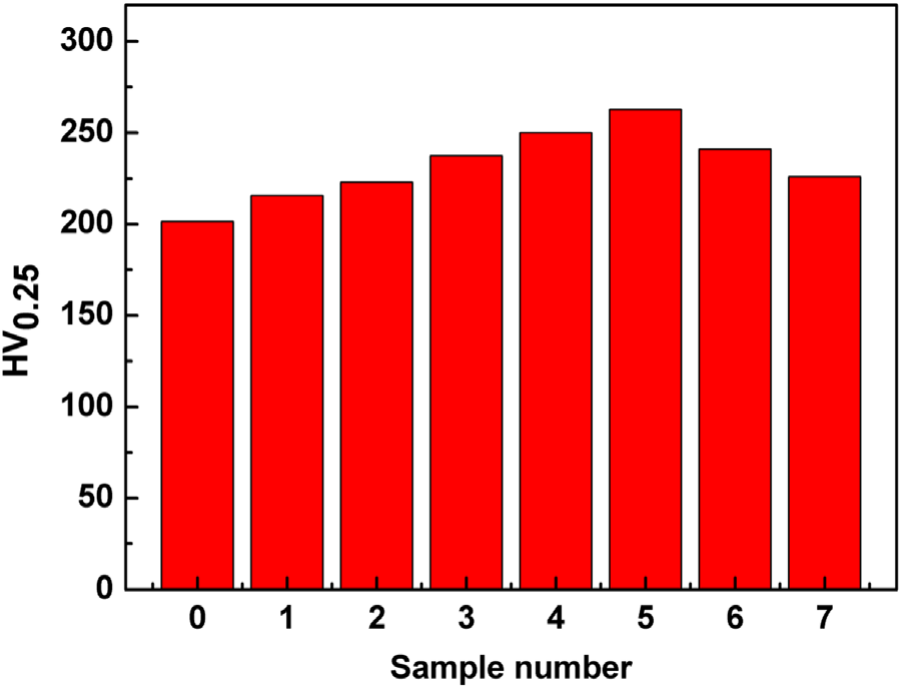
Vickers hardness of the BCSO samples: samples 0–5 stand for x=y=0, 0.01, 0.02, 0.04, 0.06, 0.08, respectively; sample 6 stands for x=0, y=0.08; sample 7 stands for x=0.08, y=0.

These data show the Vickers hardness of pristine BiCuSeO and the dispersion strengthening **[1]** effect of Ba/Pb co-doping upon it (Fig. 1).

## 2 Experimental design, materials and methods

All the specimens were polished with two parallel planes and the plane close to the central part was utilized for Vickers hardness (HV) measurement, which were conducted on HV-10008 with a load of 25 g and a loading time of 15 s. Every sample was tested with 5 data points, and the average HV was used for characterizing mechanical properties.The results are very intuitive numerical values.

## Figures and Tables

**Fig. S1:**
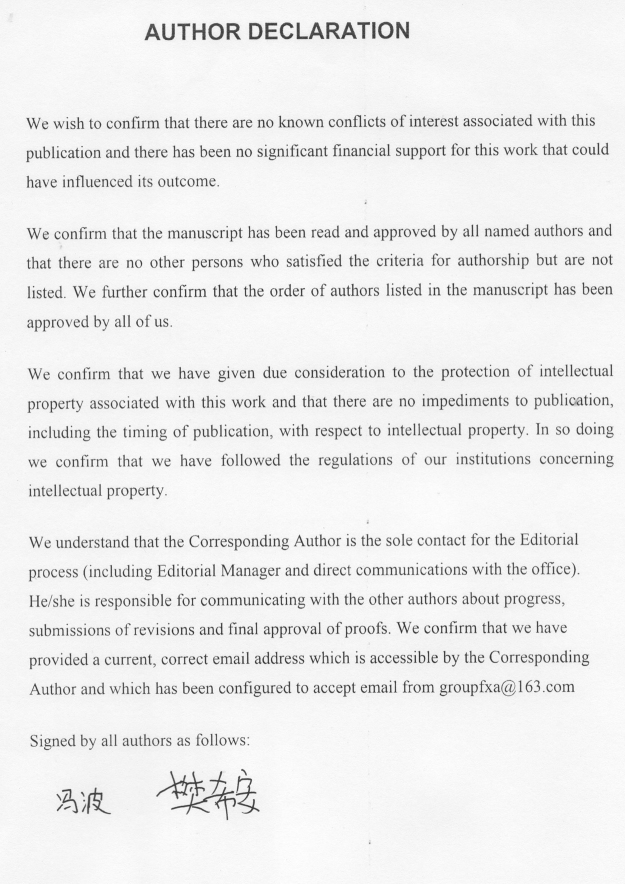
.
